# Maximizing the efficiency of decoding by selecting heterogeneous inputs from a population of electrosensory neurons responding to communication signals

**DOI:** 10.1186/1471-2202-14-S1-P261

**Published:** 2013-07-08

**Authors:** Gary Marsat

**Affiliations:** 1 Department of Biology & Center for Neuroscience, West Virginia University, Morgantown, WV 26506, USA

## 

Neural processing of sensory signals relies on spatially and temporally distributed patterns of activity across a population of neurons; understanding the structure of these patterns of activity is key to understanding neural codes and thus how information is extracted from the environment to guide specific behaviors. It has long been recognized that a population of neurons of the same type, in a given brain area and subserving a similar function, must be regarded as a synergistic whole [1]. Only in recent years has the heterogeneity of neural properties within such a population, and its critical importance to the function of the population, been fully appreciated [2,3].

In the electrosensory system of gymnotiform fish, neural heterogeneity in the primary sensory area of the hindbrain (i.e. the ELL) has been shown to enhance the accuracy of encoding of a specific kind of communication signal: big chirps. Big chirps are commonly produced by males during courtship interactions with females. As with any courtship signal, its quality could influence the female's response, and thus, detailed information about the signal's characteristics should be acquired by the nervous system. The pyramidal cells of the ELL respond to big chirp with a graded increase in firing rate, and their spiking patterns are very variable from cell to cell, much more than from one response of a cell to another response of the same cell to the same stimulus. Even cells that belong to the same sub-class can differ significantly in their response patterns. In a recent paper [4] it was shown that a simple decoder relying on the output of this heterogeneous population of cells could perform a discrimination task with more accuracy than if the input to the decoder was a population of homogeneous cells. The homogeneous response was mimicked by assembling a population response made of several responses of the same cell. In this analysis we compared two scenarios: an artificially homogeneous population input to an input made of the simple sum of randomly selected cells from the heterogeneous population. Theory predicts that the exact composition of this heterogeneous population should influence the amount of information it carries and that the composition should limit the redundancy of the information coded while still allowing for neural noise to be averaged out. Here, we aim to determine empirically the composition of the population response that would allow the decoder to most accurately perform a discrimination task.

In vivo responses of ELL pyramidal cells to several repetitions of a variety of big chirps were recorded. The response of each cell was transmitted to the decoder through a synapse of variable strength. A decoder based on a spike-train distance metric [5,6] allowed us to quantify the amount of information carried by the population input and estimate the accuracy with which two different big chirps could be discriminated. A supervised learning rule changed the weight of the synapses between each cell and the decoder thereby shaping the population input to allow more accurate discrimination. After the synapse weight reached their optimal value we characterized the composition of the population input. The learning process selected components of the population that were least correlated and that were also reliable in their spiking pattern from trial to trial. The large difference between the discrimination accuracy before and after learning has occurred suggests that such a shaping of population inputs would permit the system to perform significantly better and thus, our results show how plasticity could fine-tune a network to maximize the amount of information it carries.

## References

[B1] Georgopoulos AP Schwartz AB Kettner RE Neuronal Population Coding of Movement Direction Science 1986 233 47771 1416 1419 3749885 10.1126/science.3749885

[B2] Marsat G Longtin A Maler L Cellular and circuit properties supporting different sensory coding strategies in electric fish and other systems Curr Opin Neurobiol 2012 10.1016/j.conb.2012.01.009 22326255

[B3] Pitkow X Meister M Decorrelation and efficient coding by retinal ganglion cells Nat Neurosci 2012 15 4 628 635 10.1038/nn.3064 22406548 PMC3725273

[B4] Marsat G Maler L Neural heterogeneity and efficient population codes for communication signals J Neurophysiol 2010 104 5 2543 2555 10.1152/jn.00256.2010 20631220

[B5] Larson E Billimoria CP Sen K A biologically plausible computational model for auditory object recognition J Neurophysiol 2009 101 1 323 331 18987124 10.1152/jn.90664.2008 PMC2637019

[B6] van Rossum MC A novel spike distance Neural Comput 2001 13 4 751 763 10.1162/089976601300014321 11255567

